# A Novel Strategy to Construct Yeast *Saccharomyces cerevisiae* Strains for Very High Gravity Fermentation

**DOI:** 10.1371/journal.pone.0031235

**Published:** 2012-02-17

**Authors:** Xianglin Tao, Daoqiong Zheng, Tianzhe Liu, Pinmei Wang, Wenpeng Zhao, Muyuan Zhu, Xinhang Jiang, Yuhua Zhao, Xuechang Wu

**Affiliations:** Institute of Microbiology, College of Life Sciences, Zhejiang University, Hangzhou, Zhejiang Province, People's Republic of China; University of Nottingham, United Kingdom

## Abstract

Very high gravity (VHG) fermentation is aimed to considerably increase both the fermentation rate and the ethanol concentration, thereby reducing capital costs and the risk of bacterial contamination. This process results in critical issues, such as adverse stress factors (ie., osmotic pressure and ethanol inhibition) and high concentrations of metabolic byproducts which are difficult to overcome by a single breeding method. In the present paper, a novel strategy that combines metabolic engineering and genome shuffling to circumvent these limitations and improve the bioethanol production performance of *Saccharomyces cerevisiae* strains under VHG conditions was developed. First, in strain Z5, which performed better than other widely used industrial strains, the gene *GPD2* encoding glycerol 3-phosphate dehydrogenase was deleted, resulting in a mutant (Z5Δ*GPD2*) with a lower glycerol yield and poor ethanol productivity. Second, strain Z5Δ*GPD2* was subjected to three rounds of genome shuffling to improve its VHG fermentation performance, and the best performing strain SZ3-1 was obtained. Results showed that strain SZ3-1 not only produced less glycerol, but also increased the ethanol yield by up to 8% compared with the parent strain Z5. Further analysis suggested that the improved ethanol yield in strain SZ3-1 was mainly contributed by the enhanced ethanol tolerance of the strain. The differences in ethanol tolerance between strains Z5 and SZ3-1 were closely associated with the cell membrane fatty acid compositions and intracellular trehalose concentrations. Finally, genome rearrangements in the optimized strain were confirmed by karyotype analysis. Hence, a combination of genome shuffling and metabolic engineering is an efficient approach for the rapid improvement of yeast strains for desirable industrial phenotypes.

## Introduction

Bioethanol, a clean and renewable biofuel, is a good alternative to petrol. Global interest on fuel ethanol production increased considerably since 1970 due to the oil crises. The ethanol market yield is expected to reach 100 billion liters in 2015 [1]. Yeast *Saccharomyces cerevisiae* strains are the most exploited and primary microbes known to the industry for potable and industrial ethanol production [2]. Based on the present fermentation technology, ethanol concentration is usually among 10%–14% (v/v), when a substrate contains 180–220 g/l total sugars [3]. Nevertheless, an opportunity still exists for process improvement, which will produce more desirable production economics. Very high gravity (VHG) fermentation is such a process that allows a considerable increase in both the fermentation rate and the ethanol concentration, reducing capital costs and the risk of bacterial contamination. This process is defined as the preparation and fermentation of mashes containing 27 g or more dissolved solids per 100 g mash [4], [5]. During industrial VHG fermentation, yeast cells are exposed to several stresses including osmotic stress (resulting from the high sugar concentration at the beginning of fermentation) and ethanol stress (resulting from high concentration of ethanol at the end of fermentation) and led to stuck or slugglish fermentation [6], [7], [8]. Besides, under VHG conditions, more glycerol, which could consume up to 4% carbon source in industrial fermentations, were formed as a counterbalancing product to maintain the redox or osmotic balance of yeast cells [9]. Thus, breeding yeast strains with higher tolerance of these stresses, concomitant with less byproduct formation, is essential to improve ethanol productivity.

Studies on global gene expression have indicated that stress tolerance are complex traits under the control of multiple genes that are difficult to modify with traditional breeding, metabolic engineering, or other genetic manipulation methods [10], [11]. Due to such complexity, genome shuffling, has emerged as a whole genome engineering approach for strain improvement [12], [13]. This approach allows the improvement of complex polygenic phenotypes by combining useful genetic traits of multiple parental strains into a single strain. This strategy has been successfully applied for the rapid improvement of industrially important microbial phenotypes (ie., osmotic pressure tolerance, thermotolerance, ethanol tolerance, and ethanol productivity in *S. cerevisiae*) [14]. However, genome shuffling has limits in practice due to an insufficiency in proper screening method to control the yield of metabolic byproducts. Hou found that the shuffled strain not only enhanced ethanol productivity under VHG conditions, but also increased the glycerol productivity [15]. Although the high production of glycerol has been demonstrated to be beneficial for strain stress resistance [15], this condition undoubtedly reduces the productivity of ethanol and offsets the advantage of VHG fermentation. Over the past decade, the tools in metabolic engineering have remarkably enabled targeting of necessary genetic changes for yeast cells to express desired phenotypes. Glycerol synthesis can be controlled by this technology based on direct genetic manipulation of key genes (ie., two isoenzymes of glycerol 3-phosphate dehydrogenase, namely, *GPD1* and *GPD2*) involved in glycerol metabolism. Nevertheless, most commonly, genetic manipulation (ie., *GPD1* and *GPD2* deletion) may negatively affect strain performance [16]. Therefore we adopted an approach combing the two aforementioned methods.

In the present study, the fermentation capacities of some commonly used bioethanol yeast strains were initially compared. Among these strains, the best performing strain, Z5, was chosen as the original strain for VHG fermentation performance improvement. To lower the glycerol concentration and raise the rate of sugar-to-ethanol conversion of recombinants, gene *GPD2* involved in the glycerol synthesis was knocked out in strain Z5. Then, genome shuffling was used to further improve the fermentation performance of the engineered strain Z5Δ*GPD2*. After three rounds of genome shuffling, recombinant SZ3-1, which showed significantly improved fermentation capacity than Z5, was selected. This improvement was mainly due to the enhancement of ethanol tolerance in the shuffled strain, which is tightly associated with cell membrane compositions and trehalose accumulations. These results demonstrate that the novel strategy proposed in this study is effective in improving the ethanol production performance of industrial *S. cerevisiae* strains under VHG conditions.

## Materials and Methods

### Strains and media

Yeast strains Z0 (CICC 1300), Z1 (CICC 1308), Z2 (CICC 1043), Z3 (Angel active dry yeast), Z4 (*S. cerevisiae var. llipsoideus*), and Z5 (mutant from strain Z4) were industrial strains widely used in ethanol production. Strains Z5Δ*GPD2* and Z5*TPS1-2* was respectively constructed by deleting the *GPD2* gene and overexpressing *TPS1* and *TPS2* in stain Z5. Strain SZ3-1 was the strain newly constructed in the current study.

Growth medium YPD contained 10 g/l yeast extract, 20 g/l peptone, and 20 g/l glucose. The SD medium contained 6.7 g/l yeast nitrogen base without amino acids and 20 g/l glucose. A fermentation medium from corn mash was prepared by double enzyme hydrolysis [17].

### *GPD2* knockout

Plasmid DNA from *Escherichia coli* and genomic DNA from *S. cerevisiae* were obtained by using Plasmid Mini Kit (Omega Bio-tech, USA) and Yeast DNA Kit (Omega Bio-tech, USA), respectively. DNA primers were purchased from GenScript Inc. (Nanjing, China).

Strain Z5Δ*GPD2* was obtained by a one-step disruption of *GPD2*. The *GPD2*-*kanMX* disruption cassettes contained, from left to right, fragment GPD2U (the nucleotides -687 to -4 upstream of the ATG start codon of *GPD2*), the *kanMX* gene, and fragment GPD2D (the nucleotides 22 to 693 downstream of *GPD2*). *E. coli* Top 10 and *S. cerevisiae* cells were transformed as described earlier [18], [19]. Transformants were selected from the YPD medium supplemented with 300 µg/ml G418. Correct deletion of *GPD2* was verified by PCR analysis using a combination of corresponding target gene-specific primers (Table S1). To eliminate the G418-resistant gene from the successfully disrupted genome, the target mutant was transformed with the Cre recombinase expression plasmid pSH65 [20]. *GPD2*-*kanMX* disruption cassettes were repeatedly used to completely deleted *GPD2* until no PCR product emerged using primers GPD2S and GPD2A (Table S1).

### *TPS1* and *TPS2 overexpression*

To overexpress the genes *TPS1* and *TPS2*, their ORF were amplified by PCR and then cloned into the *Bam*HI and *Xho*I sites behind the PGK promoter of the pYES2-derived plasmids pYES3 (contains gene *ble* ^r^) and pYKS3 (contains gene *kan^r^*), respectively. Subsequently, plasmids pYES3TPS1 and pYKS3TPS2 was introduced into strain Z5 and the transformants were selected on YPD plate containing 50 µg/ml zeocin and 300 µg/ml G418.

### Genome shuffling

The best performing strain was selected after three successive rounds of sporulation and hybridization. In the first round, strain Z5 freshly harvested from the YPD medium was grown on the sporulation medium for 5–7 days. The cells were subsequently collected and washed thrice with sterile water, followed by isolation of spores and protoplast regeneration. The resulting cells were mated randomly and adequately in the YPD liquid medium for about 24 h. Afterward, these hybrids were appropriately diluted and spread on selective plates (for details, see ref [21]), and then fast growing colonies were selected and tested. Finally, hybrids with good fermentation capacities selected from the first round served as the starting strains for the subsequent rounds of genome shuffling, which were conducted using the same methods. The fermentative stability of ultimately selected hybrids was also determined by analyzing fermentation performance of these hybrids and karyotypes every 10 generations after successive subcultures on the YPD medium for 50 generations.

### Fermentation and metabolites

The yeast cells were precultured in 5 ml YPD medium in a test tube at 30°C without shaking for 24 h and then transferred totally into corn mash in an Erlenmeyer flask containing approximately 160 g/l total sugars for cultivating at 30°C for 12–16 h with shaking at 200 r/min. Yeast cells were then harvested and inoculated in a fermentation medium (200 g corn mash, 280 g/l total sugars, and pH 5.0) at a concentration of 1×10^5^ cells/ml. Anaerobic fermentation was performed in 500 ml Erlenmeyer flasks with fermentation locks for 72 h. The concentrations of main fermentation metabolites were measured on an Aminex HPX-87H column (Bio-Rad) at 60°C [22].

### Cell viability and membrane integrity

Yeast cells from the fermentation broth were collected at intervals by centrifugation and were then appropriately diluted in plates on the YPD medium. Cell viability was analyzed by calculating the colony-forming unit that emerged in each plate.

Cell membrane integrity during ethanol fermentation at different fermentation periods was examined by fluorescent staining with propidium iodide (PI) and fluorescein diacetate (FDA). Yeast samples from the fermentation broth were harvested, washed, and resuspended in 1×PBS (pH 7.2) to a final OD_600_ of 0.1 (about 1×10^6^ cells). A 10 µl PI stock solution in 1×PBS (500 µg/ml) and FDA stock solution in acetone (1 mg/ml) were added to 100 µl cell suspensions just prior to the staining and were then gently vortexed for staining in the dark for 30 min. Fluorescence detection was performed by LSM-510 (Zeiss, Germany). Three fields of view from each cover slip were randomly chosen.

### Measurement of diffusion of intracellular nucleotide

Early-stationary-phase cells were harvested and washed until the absorbance of the supernate at 260 nm was negligible. The cells were suspended in 0%, 10%, 15%, and 20% (v/v) ethanol and incubated at 30°C. After low-speed centrifugation to remove cells, the absorbances of supernates at 260 and 280 nm were measured every 3 h until they reached equilibrium. The calculating equation is as follows [23]:
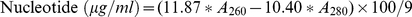



### Fatty acid and ergosterol analysis

Yeast cells were cultivated in the YPD medium at 30°C with shaking at 200 r/min for 20 h. Then, the cells were harvested and transferred to the SD medium with and without 10% ethanol. After cultivating for 24 h, total fatty acids and sterols were extracted as previously described [24]. The composition of fatty acids was analyzed by gas chromatography with a GC FOCUS, equipped with a DSQ II MS detector (Thermo, USA) on a DB-5 MS capillary column (J&W Scientific Inc., Folson, CA, USA). The operation conditions were as follows: hold at temperature 140°C for 2 min, then from 140 to 170°C at 4°C/min, hold at 170°C for 1 min, from 170 to 240°C at 3.5°C/min, hold at 240°C for 12.5 min, from 240 to 260°C at 12°C/min, hold at 260°C for 2 min; injector temperature: 250°C; MS Transfer Line temperature: 250°C; ion source: 250°C; carrier gas: helium; carrier gas flow: 1.0 ml/min; injection volume: 1 µl. Fatty acid composition was calculated based on the area of each peak. Ergosterol content was measured using the HPLC system equipped with a reverse-phase column [22] and expressed as mg ergosterol per g dry weight. Samples for dry weight analysis were washed with sterile water and then dried at 100°C overnight.

### Trehalose and enzymatic activity determination

Yeast cells were precultivated in the SD medium at 30°C with shaking at 200 r/min for 20 h. The cells were harvested and subjected to ethanol treatment for 2 h. Trehalose content in the yeast cells after exposure to 0%, 5%, 10%, and 15% (v/v) ethanol was determined using the anthrone method [25].

Cell-free extracts of yeast cells treated with 0% and 10% (v/v) ethanol for enzyme assays were obtained using the Yeastbuster protein extraction reagent (Novagen, Germany). Trehalose-6-phosphate synthase (Tps1) and trehalase activities (Ath1 and Nth1) were determined as reported previously [26]–[29]. One unit of Tps1 activity was defined as the amount of enzyme that produces 1.0 µmol of NAD^+^ per minute at 37°C and pH 6.6 [26], [29]. Glucose concentration in the supernates was measured using the glucose oxidase/peroxidase assay. Specific activity of trehalase was expressed as nmol of glucose liberated per min per mg total protein [27], [28]. Total protein concentration was measured by the method of Bradford [30].

### Quantitative RT-PCR

Total RNA was extracted from yeast cells cultivated in the SD medium with or without ethanol (10%) using the Fungal RNAout kit (TIANDZ, Beijing) according to the manufacturer's instructions. RNA samples were reverse transcribed into cDNA using the PrimerScript RT reagent Kit With gDNA eraser (TaKaRa, Japan).

Quantification of *TPS1*, *TPS2*, *TPS3*, *TSL1*, *ATH1* and *NTH1* RNA levels were quantified by quantitative RT-PCR using an ABI Prism 7500 StepOnePlus instrument (Applied Biosystem). Study samples were tested in triplicate in a 96-well plate (Axygen, USA) with a final volume of 20 µl. Primers used for quantitative PCR (Table S2) were designed using Primer Premier 5.0 software. After completion of the PCR cycles, melting curve data were then collected to verify primer specificity. DNA dilution series were prepared to calculate the amplification efficiency coefficient for each primer pair with the sample cDNA as the template. The relative expression of genes was quantified using the comparative 2^−ΔΔCT^ method with *ACT1* as the reference gene [31].

### Pulsed field gel electrophoresis (PFGE)

Yeast cells were cultivated in the YPD medium at 30°C for 48 h to reach the late stationary phase. DNA for electrophoretic karyotyping was prepared in an agarose plug, as described by Argueso [32]. PFGF was performed with a CHEF Mapper XA apparatus (CHEF Mapper XA; Bio-rad Laboratories, Hercules, CA) using the chromosomes of the *S. cerevisiae* strain BY4743 as the standard markers. Yeast chromosomes were separated on 1% pulsed-field-certified agarose gel (Bio-rad Laboratories, Hercules, CA) as follows: 24 h at 6 V/cm for 60–120 s with a time ramp at an included angle of 120°. The running buffer used was 0.5×TBE cooled at 14°C.

## Results

### Screening of the parent strain for further breeding

To select the original strain for further breeding, comparisons of VHG fermentation performances of strains Z0, Z1, Z2, Z3, Z4, and Z5 were conducted. Strain Z5 was the most suitable original strain for further breeding due to its higher ethanol yield and rate of sugar-to-ethanol conversion than those of other strains (Table 1). However, the fermentation capacity of strain Z5 (such as residual sugar) required further improvement to achieve the industrial standard (<2 g/l).

**Table 1:** Fermentation performance comparisons between industry strains under VHG conditions.

Strain	Fermentation products (g/l)			Residual glucose(g/l)	FermentationRate(g/l/h)	Rate of sugar-to-ethanol conversion
	Ethanol	Glycerol	Acetate			
Z0	108.15±1.06	11.51±0.09	0.36±0.05	36.75±1.44	1.50±0.01	0.445±0.002
Z1	115.50±1.14	11.99±0.17	0.64±0.10	22.14±1.50	1.60±0.02	0.448±0.002
Z2	103.73±1.12	11.34±0.28	0.94±0.03	46.04±1.31	1.44±0.02	0.443±0.002
Z3	112.10±1.89	12.26±0.24	0.54±0.04	30.71±1.51	1.56±0.03	0.450±0.005
Z4	115.74±0.75	11.83±0.37	0.48±0.04	21.36±1.28	1.61±0.01	0.447±0.002
Z5	120.63±2.38	10.53±0.32	0.74±0.02	14.38±1.33	1.68±0.03	0.454±0.006
Z5Δ*GPD2*	115.99±2.51	8.43±0.29	0.36±0.03	29.24±3.12	1.61±0.02	0.462±0.003

Data are the mean values and standard deviation of three dependent experiments.

### A new breeding strategy to improve the fermentation performance of strain Z5

First, the key gene *GPD2* involved in glycerol synthesis was deleted from strain Z5, resulting in strain Z5Δ*GPD2*. As speculated, the glycerol yield of strain Z5Δ*GPD2* decreased by 20% after *GPD2* deletion compared with parent strain Z5 (Table 1), but to a certain extent, final ethanol yield was also affected (4% less than that of Z5). However, the Z5Δ*GPD2* strain had higher rate of sugar-to-ethanol conversion than Z5 [16]. The contradicting results might be due to the incomplete fermentation of the Z5Δ*GPD2* strain. As previously stated, the strain with deletion of *GPD2* had a delayed response to glucose consumption and ethanol production [16], thus a lower fermentation rate.

**Figure 1:**
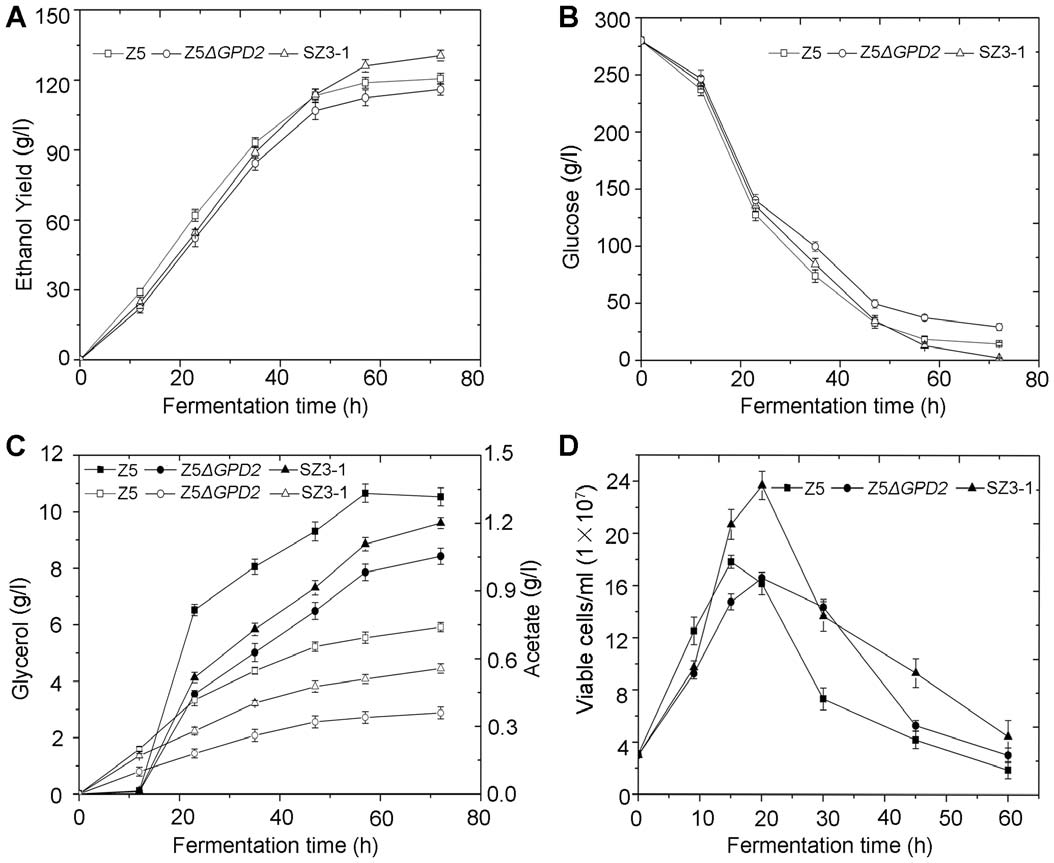
The fermentation performance of strains Z5, Z5Δ*GPD2* and SZ3-1. The ethanol yield (A), glucose consumption (B), glycerol (filled symbols) and acetate (open symbols) production (C), and cell survival rate (D) of control strains Z5 (squares), Z5Δ*GPD2* (circles), and shuffled strain SZ3-1 (triangles) during fermentation were monitored and compared.

**Figure 2:**
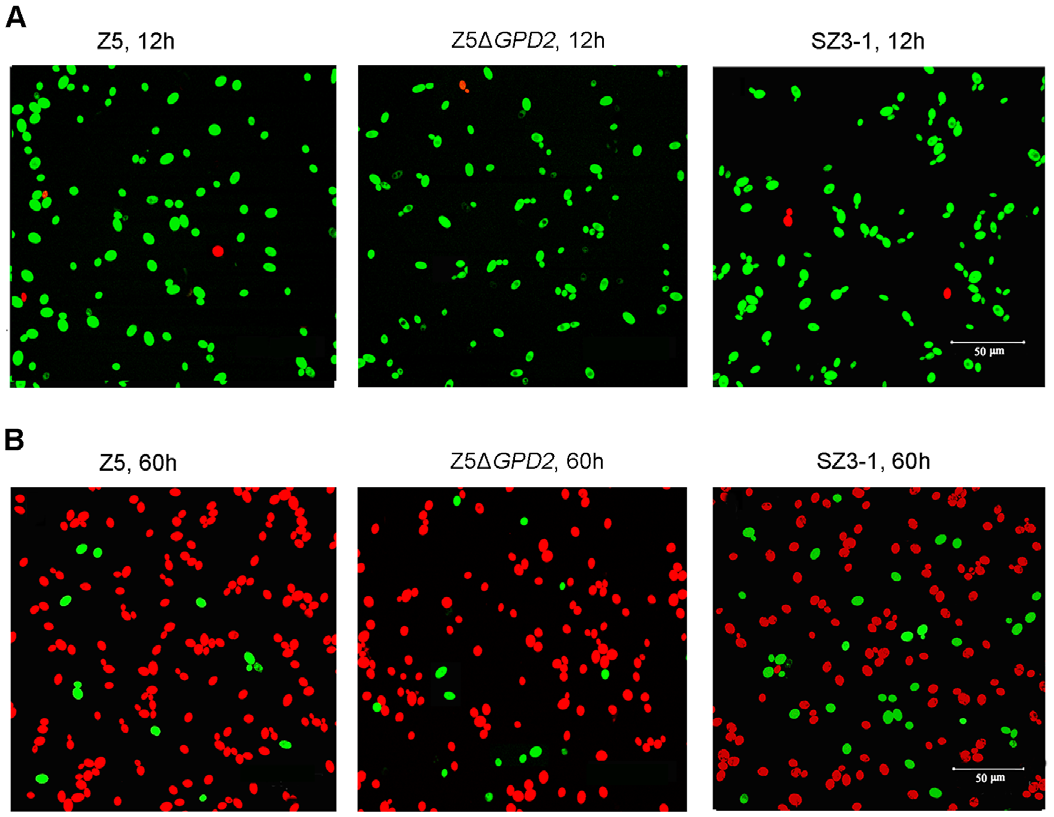
Cell membrane integrity of strains Z5, Z5Δ*GPD2* and SZ3-1 at different time of fermentation. Yeast cells harvested at 12 h (A) and 60 h (B) of fermentation, respectively, were stained with PI and FDA. Viable cells were stained green with FDA and the cells that had lost membrane integrity were stained red with PI.

Strain Z5Δ*GPD2* was then used as the starting population for genome shuffling in the current study. After each round of genome shuffling, 300 fast growing colonies were picked from the selecting plates, and 10 mutants with desired properties based on fermentation comparisons were selected and pooled to the next round of genome shuffling. Finally, after three rounds of genome shuffling, the strain (namely, SZ3-1) that performed best among the 300 shuffled strains from the third round and with good fermentative stability was selected for further study. The results of VHG fermentation showed that the fermentation capacity of strain SZ3-1 had been considerably improved compared with those of the control strains Z5 and Z5Δ*GPD2* (Figure 1). At the end of fermentation, strain SZ3-1 nearly consumed all residual sugars in the fermentation broth and enhanced the ethanol yield by 8% compared with strain Z5 (Figure 1A and 1B). Strain SZ3-1 exceeded strain Z5 in ethanol yield and glucose consumption mainly between 48 and 72 h. Compared with SZ3-1, the cell viability of control strains Z5 and Z5Δ*GPD2* dropped drastically in the later fermentation phase (Figure 1D), and more petite colonies (respiratory deficient cells) emerged (data not shown). Moreover, strains Z5 and Z5Δ*GPD2* exhibited inferior cell membrane integrity compared with strain SZ3-1 in the later fermentation phase, but without obvious differences in the earlier phase (Figure 2). Thus, strain SZ3-1 probably possesses a more prominent ability to resist the adverse environmental stresses in the later fermentation phase.

### Enhanced performance of strain SZ3-1 compared with Z5 and Z*5*Δ*GPD2* in ethanol tolerance

**Figure 3:**
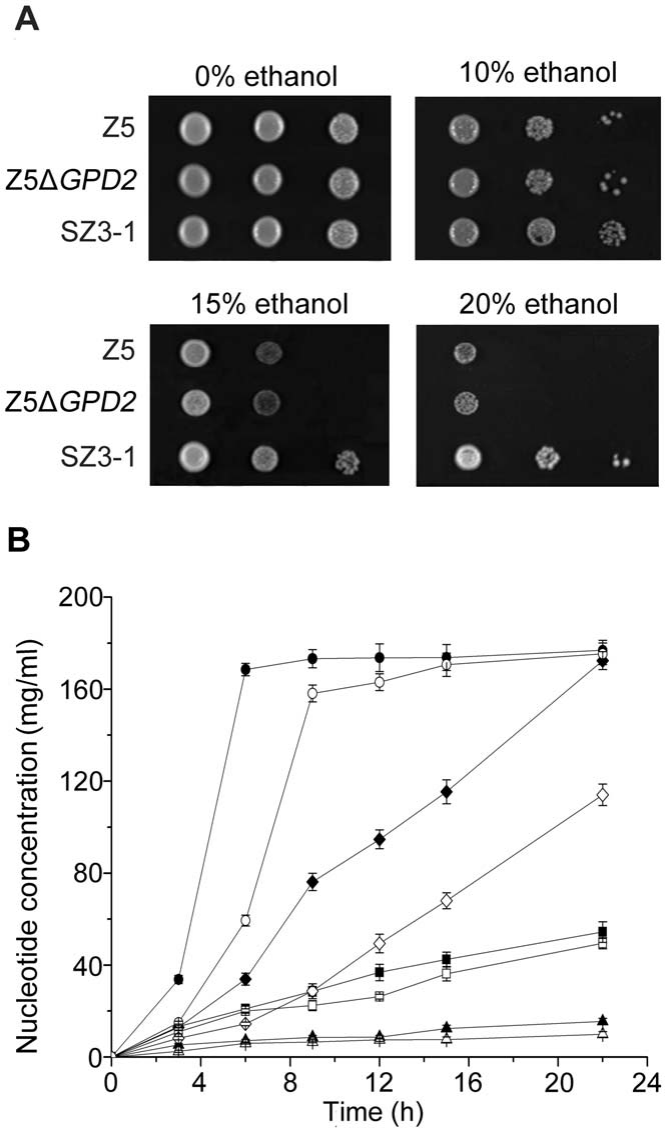
Determination of ethanol stress tolerance of strains Z5, Z5Δ*GPD2* and SZ3-1. (A) Growth of strains Z5, Z5Δ*GPD2* and SZ3-1 on different concentration of ethanol. Cells were grown in the YPD liquid medium at 30°C overnight and 10-fold serial dilutions of each sample were spotted onto the YPD medium and the YPD medium containing 10%, 15%, 20% (v/v) ethanol. Stress tolerance was calculated as the percentage of biomass formation or viable cells in stressed culture compared with that in control culture. (B) Time course of extracellular nucleotide concentration in cell suspension of strains Z5 (filled symbols) and SZ3-1 (open symbols). Yeast cells were suspended in aqueous solution with 0% (triangles), 10% (squares), 15% (diamonds), and 20% (circles) (v/v) ethanol and incubated at 30°C. Concentration of nucleotide that leaked into the supernatant was measured every few hours.

During ethanol fermentation, the increasing concentration of ethanol could gradually reduce cell viability mainly by influencing the integrity of the cell membrane and its function [33], [34]. As illustrated by Figure 3A, the ethanol stress tolerance of strain Z5 was inferior to that of shuffled strain ZS3-1 but similar to that of Z5Δ*GPD2* strain. When subjected to ethanol, the nucleotide that leaked into the supernate of strain SZ3-1 was always less than that of Z5 (*P*<0.05) and the differences widened with the increase of ethanol concentration (the data of strain Z5Δ*GPD2* was similar to those of strain Z5 but not shown; Figure 3B). These results illustrated that strain SZ3-1 had better capability to maintain cell membrane integrity under ethanol stress than that Z5, indicating the possible mechanisms for the improved ethanol stress tolerance of strain SZ3-1.

### Relationship between ethanol tolerance and cell membrane composition

Under the ethanol stress, yeast cells may change membrane compositions to confront membrane fluidization and stabilize the plasma membrane [35]. Among the various membrane components, unsaturated fatty acid and ergosterol were considered as the two critical determinants of ethanol tolerance [36].

The main fatty acids of yeast cell membranes are divided into saturated fatty acids (palmitic acid C_16:0_ and stearic acid C_18:0_) and unsaturated fatty acids (palmitoleic acid C_16:1_ and oleic acid C_18:1_) [6]. When grown in the absence of ethanol, strain SZ3-1 had markedly higher proportions of C_18_ fatty acids, especially C_18:1_, and a slightly lower unsaturation index than that of strain Z5. In terms of unsaturated fatty acids, strain Z5 and strain SZ3-1 were indistinguishable. After exposure to 10% ethanol, both strains had a further increase in C_18_ fatty acid total content (8% and 9% for strain Z5 and SZ3-1, respectively), concomitant with a dramatic decline in C_16_ fatty acid, whereas the concentration of unsaturated fatty acids and the unsaturation index remained relatively constant. However, both increments of total C_18_ fatty acid proportions and C_18:1_ fatty acid proportions in strain SZ3-1 were higher than those of Z5.

Ergosterol also plays a critical role on ethanol stress tolerance in *S. cerevisiae* by stabilizing the normal structure of membranes [37]. The biosynthesis of ergosterol was slightly reduced in the presence of ethanol (Table 2). However, contrary to what had been observed for fatty acids, the concentrations of ergosterol in strains Z5 and SZ3-1 were barely different whether confronted with ethanol or not.

**Table 2:** Fatty acid compositions in plasma membrane and ergosterol content of strains Z5 and SZ3-1 cultivated in different conditions.

Strain	Fatty acid composition (%)^a^					Unsaturation Index (Δ/mol)^b^	ergosterol (mg/g) dry weight)
	C16:0	C18:0	C16:1	C18:1	C18:2		
Z5 (0%ethanol)	7.91	3.10	48.84	39.85	0.31	0.89	5.99
SZ3-1 (0%ethanol)	8.67	4.48	41.44	45.40	0.01	0.87	6.06
Z5 (10%ethanol)	6.88	3.93	46.37	42.54	0.27	0.89	5.19
SZ3-1 (10%ethanol)	8.58	4.86	36.98	48.61	0.91	0.86	5.25

Data are the mean values and standard deviation of three dependent experiments.

a Fatty acids are denoted by the number of carbon atoms: number of unsaturated linkeages.

b Unsaturation Index(Δ/mol) was calculated as: Δ/mol = [1×(% monoene)+2×(% diene)+3×(% triene)]/100.

### More trehalose accumulation in strain SZ3-1

**Figure 4:**
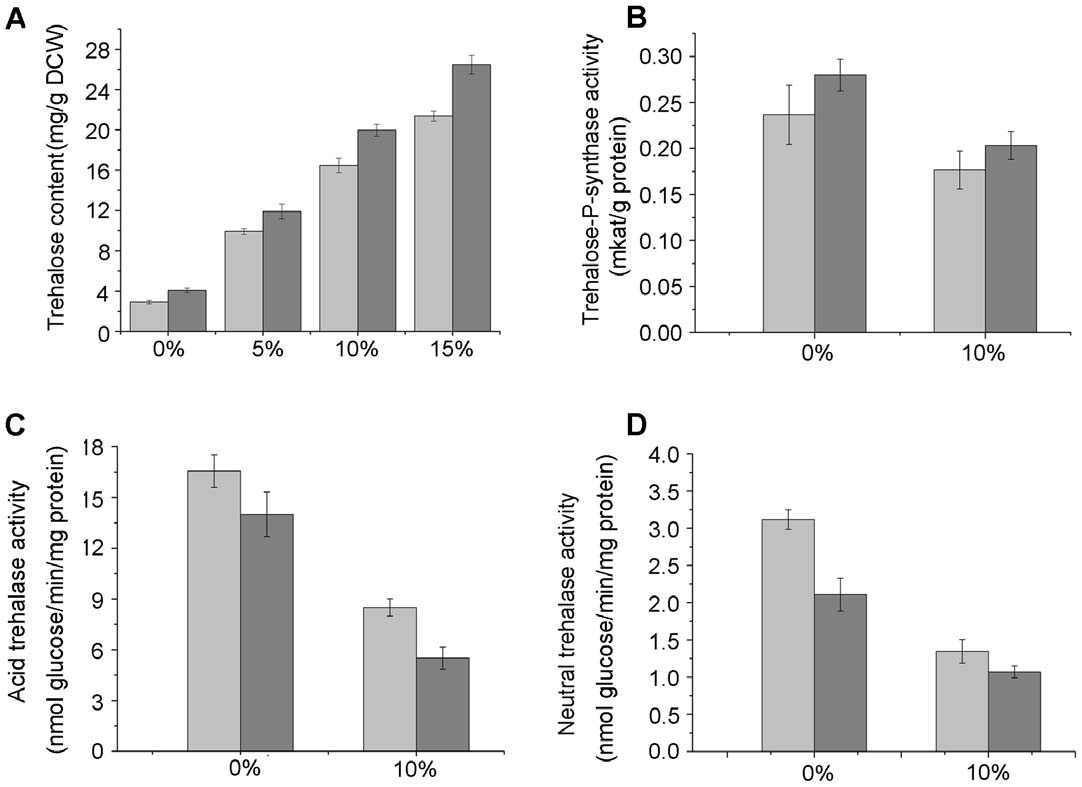
Trehalose concentrations and related enzymatic activity of stains Z5 and SZ3-1. Yeast strains were harvested at the stationary phase and exposed to ethanol stress. Trehalose concentration (A) of strains Z5 (light gray) and SZ3-1 (gray) subjected to different ethanol stress (0%, 5%, 10%, and 15% (v/v) ethanol) was measured. Finally, we determined related enzymatic activities, namely, trehalose-P-synthase (B), acid trehalase (C), and neutral trehalase (D) under 0% ethanol and 10% ethanol conditions.

A strong correlation between trehalose content and stress resistance has been revealed for a variety of stresses, especially ethanol stress [38]. Figure 4A shows that the amount of trehalose in strain SZ3-1 grown in control cultures was 29% higher than that of Z5. In the presence of 5%, 10%, and 15% ethanol, trehalose synthesis of both strains were strongly stimulated, but strain SZ3-1 still accumulated more trehalose compared with Z5. These results indicate that yeast cells accumulate trehalose as a protectant under ethanol stress.

The intracellular level of trehalose in *S. cerevisiae* is the result of a well-regulated balance between enzymatic synthesis and degradation. Figures 4B–4D show that strains Z5 and SZ3-1 displayed different enzymatic activities of both Tps1 and trehalase when cultivated in the absence or presence of ethanol. Under nonstressful conditions, strain SZ3-1 had a higher Tps1 activity (22%, Figure 4B) and lower trehalase activity (15% and 32% for acid trehalase and neutral trehalase respectively, Figures 4C and 4D) compared with Z5. When subjected to 10% ethanol, a decrease in both Tps1 (25% and 27% for Z5 and SZ3-1, respectively) and trehalase activity (49% and 61% in acid trehalase for Z5 and SZ3-1, respectively, and 57% and 49% in neutral trehalase for Z5 and SZ3-1, respectively) was observed. However, strain SZ3-1 always showed higher Tps1 activity and lower trehalase activity (including acid and neutral trehalase) than those of strain Z5 with or without ethanol (*P*<0.05). This result could precisely explain why SZ3-1 accumulated more trehalose than did Z5. Similarly, more trehalose accumulation in stressed cultures than control cultures was presumably due to the predominant role of trehalose synthesis over that of trehalose degradation (Figure 4B–4D).

**Figure 5:**
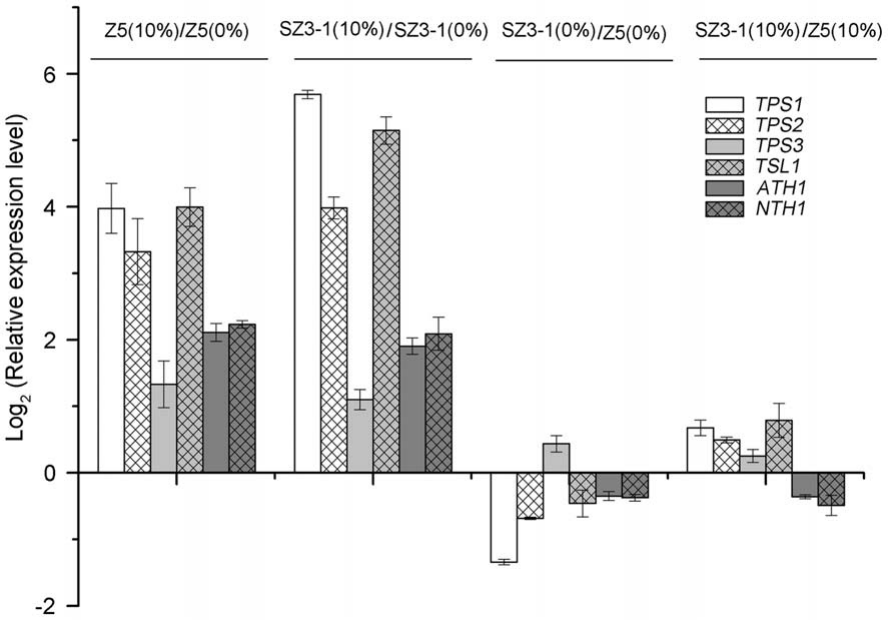
Relative expression level of six genes related to trehalose metabolism. Strains Z5 and SZ3-1 were grown in the absence (0%) or presence (10%) of ethanol. Gene expression of four cases were compared: strain Z5 grown in the presence and absence of ethanol (Z5 (10%)/Z5 (0%)); strain SZ3-1 grown in the presence and absence of ethanol (SZ3-1 (10%)/SZ3-1 (0%)); strain SZ3-1 and Z5 grown in absence of ethanol (SZ3-1(0%)/Z5(0%)); and strain SZ3-1 and Z5 grown in the presence of ethanol (SZ3-1 (10%)/Z5 (10%)).

Studies on various microorganisms have shown that trehalose accumulation induced by numerous forms of stresses is mainly mediated at the transcription level [27]. In agreement with the result that yeast cells accumulated more trehalose under ethanol stress, the expression levels of *TPS1*, *TPS2*, *TPS3*, and *TSL1*, which were involved in trehalose synthesis, were all highly upregulated in both Z5 and SZ3-1 strains after exposure to 10% ethanol. Besides, *ATH1* and *NTH1*, which encode acid trehalase and neutral trehalase, respectively, were also upregulated (Figure 5). The fact that ethanol stress induced genes involved in both trehalose synthesis and degradation might enable the yeast cell to adjust its trehalose content rapidly to counteract ethanol-induced change. In accordance with the result of enzymatic activity determinations, the upregulation of genes in the synthetic pathways and the downregulation of genes in the degraded pathway caused more trehalose accumulation in strain SZ3-1 in response to ethanol stress compared with strain Z5.

To further demonstrate the contribution of trehalose to the improved ethanol tolerance of SZ3-1, we overexpress the genes *TPS1* and *TPS2* in strain Z5 (resulting in strain Z5*TPS1-2*). The intracellular trehalose of strain Z5*TPS1-2* was significant higher than that of strain Z5 (*P*<0.05) and close to that of SZ3-1 with or without ethanol stress (Figure S1). Notably, engineered strain Z5*TPS1-2* enhanced the viability by 40% compared to Z5 under the treatment of 15% ethanol for 10 h, but still 25% less than that of SZ3-1. These results suggested (i) more intracellular trehalose indeed contributed to the ethanol tolerance of Z5 and (ii) the improved ethanol stress of SZ3-1 was the result of the changes of multiple physiological factors.

### Chromosomal rearrangement and genetic stability estimate

**Figure 6:**
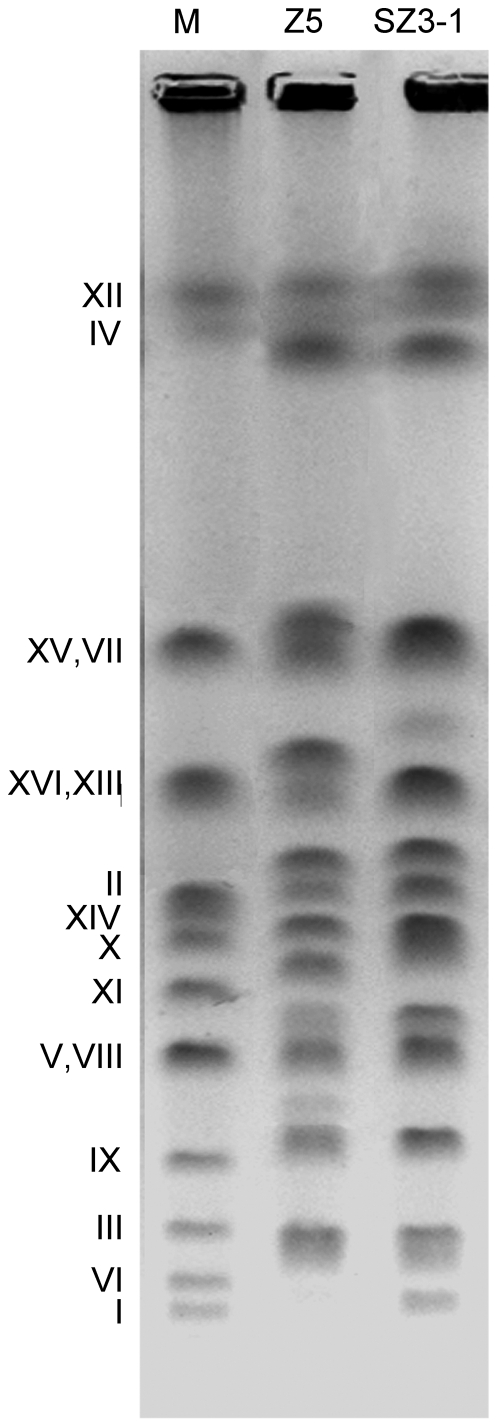
Karyotype profiles of strains Z5 and SZ3-1 obtained by PFGE. Lanes M, Z5 and SZ3-1 respectively represent the chromosomal profiles of strains BY4743, Z5 and SZ3-1. Numbers corresponding to each band are designated in the left.

PFGE was performed to determine the electrophoretic karyotype of each strain. The intact chromosomes isolated from strains Z5 and SZ3-1 were shown in Figure 6. As expected, the karyotypes of shuffled strain SZ3-1 remarkably differed from that of parent strain Z5 with the disappearance of wild-type bands and emergence of novel bands. The relative DNA content of strain SZ3-1 was slightly less than that of the original strain Z5 (Figure S2). These results suggested that chromosomal rearrangements, such as gene local amplification, chromosome copy number changes, and intrachromosomal and interchromosomal translocations, might occur in the whole genome during genome shuffling. Correspondingly, the recombination events would facilitate the combination of beneficial mutations and confer ethanol resistance in the shuffled strains. The genetic stability of the recombinant SZ3-1 was also analyzed. After successive subcultures in the YPD liquid medium for 50 generations, the karyotypes of the strain SZ3-1 from every 10 generations were determined, with the results showing that this character of this strain could be steadily inherited (data not shown).

## Discussion

The application of VHG fermentation for bioethanol production can considerably improve the final ethanol concentration and save the energy consumption. However, this technology will impose severe environmental stresses on yeast cells, often resulting in stuck fermentation and more formation of metabolic byproducts. Therefore, enhancing the stresses resistance and lowering the metabolic byproducts would be useful to improve VHG fermentation performance of yeast strains.

Genome shuffling is a recently developed approach and has been proved effective for the improvement of complex phenotypes in microorganisms [12]. However, the application of this approach is limited in the absence of an appropriate screening method to control the yield of metabolic byproducts. With the aid of metabolic engineering, this limitation can be overcome. Glycerol yield can be reduced through metabolic engineering by the deletion of the *GPD2* gene. Given that the deletion of *GPD2* affects the fermentation rate and ethanol yield, the present study proposes the performance of metabolic engineering prior to genome shuffling, considering that negative effects resulting from genetic manipulation could be circumvented during the process of genome shuffling. A mutant strain, SZ3-1, was obtained using this strategy. This strain could effectively ferment 280 g/l glucose within 72 h while simultaneously maintaining a high fermentation rate and a low glycerol yield. Through ethanol tolerance and membrane integrity analysis, strain SZ3-1 was found to show markedly enhanced ethanol tolerance than that of Z5, which contributed to its improved fermentation performance under VHG conditions.

More ethanol-tolerant strain SZ3-1 incorporated more long-chain fatty acids (mainly C_18:1_) into the membrane phospholipid than the less-tolerant strain Z5 at the expense of short-chain fatty acids. This finding is well suited with the results of Chi [39] but does not correlate with the results of Castillo Agudo [40] who have showed that the more ethanol-tolerant strains contain a lower amount of long-chain fatty acids compared with less ethanol-tolerant strains. This discrepancy might be caused by the differences in the yeast strains and the analytical procedures used. Several authors have proposed that the ability of cells to increase the proportion of unsaturated fatty acids in plasma membrane is the principal mechanisms used by yeast to adapt to the presence of ethanol [35], [41]. Surprisingly, in the current study, the proportion of total unsaturated fatty acids varied very slightly between the two strains, similar to the degree of fatty acid unsaturation.

Ergosterols represent another category of lipid components in the yeast membrane that is responsible for structural membrane features. Castillo Agudo [40] found yeast strains with the highest concentration of ergosterol to be the most tolerant of ethanol. In the present study, no difference was observed in the ergosterol levels between strain Z5 and SZ3-1, even exposed to ethanol. In agreement with the repressive effect of ethanol on ergosterol biosynthesis [35], [42], the ergosterol concentrations of these two strains were decreased after ethanol treatment.

As a protectant that contributed to the survival of yeast under various stressful conditions, more trehalose accumulation was observed in strains Z5 and SZ3-1 under ethanol stress. Corresponding with previous studies [38], [43], strain SZ3-1 always had higher concentration of intracellular trehalose (Figure 4A) and could better coped with ethanol stress with greatly increased trehalose content than the strain Z5. The increasing intracellular trehalose content was probably a mechanism for yeast cells to respond to ethanol stress, and the difference in the trehalose content in each strain was the key to the diverse tolerance of ethanol stress. A decrease in both trehalose synthase and trehalase was observed in yeast cells after ethanol exposure. Nevertheless, several genes associated with trehalose metabolism were highly expressed (*TPS1*, *TPS2*, *TPS3*, *TSL1*, *ATH1*, and *NTH1*), as reported by Li and Kaino [38], [43]. The conflicting result might be due to the adverse effect of ethanol on the enzyme structure [44]. Ethanol might affect the activity of trehalose synthase and trehalase at different extents. Thus, more trehalose accumulation in stressed cultures than control cultures was due to the predominant role of trehalose synthesis over the role of trehalose degradation (Figure 4B–4D and 5). In particular, the superior role of trehalose synthesis in strain SZ3-1 was more obvious than that of strain Z5. Overall, variations in the cell membrane components and trehalose are considered to be important determinants of ethanol tolerance in the shuffled strain.

Ethanol stress tolerance is a complicated phenotype controlled by multiple genes and is difficult to be altered by single gene modification. Improvements in ethanol tolerance, as well as relevant physiological and biochemical characteristics, indicated that large-scale genomic changes occurred in yeast strain SZ3-1. The PFGE result revealed and confirmed the occurrence of the gross chromosomal rearrangements during the process of genome shuffling considering that strains Z5 and SZ3-1 displayed quite different karyotype profiles, with differences in both chromosome lengths and chromosome numbers. Chromosomal rearrangements were considered to play an important role in yeast evolution and adaptation [45]. In the present study, through successive chromosome rearrangements during sporulation and hybridization, ethanol tolerance of strain SZ3-1 was improved by adjusting cell membrane compositions and trehalose concentrations. These results further prove the effectiveness of genome shuffling in the modification of the regulatory of multiple metabolic pathways and complex phenotypes.

To the best of the author's knowledge, the present study is the first report to introduce the combination of genome shuffling and metabolic engineering into industry breeding for industrial yeast strains. The proposed novel technique has been proved effective in enhancing the fermentation performance under VHG conditions of yeast strains in the current study. The technique could not only reduce the yield of the undesired product that is inevitably generated during fermentation and improve the yield of the target product, but also improve the complex phenotypes, which are difficult to modify by traditional approaches. This proposed strategy could also be applied on other trait improvements or on other microorganisms. The authors expect that the strategy developed in the present study can be used as an efficient tool for industrial strain breeding.

## Supporting Information

Figure S1 Trehalose content and cell viability of strain Z5, Z5*TPS1-2*, SZ3-1 under 15% ethanol stress.
(DOC)

Figure S2 Comparison of DNA content of strains Z5 (red) and SZ3-1 (black) using flow cytometry.
(DOC)

Table S1 Primers used for *GPD2* deletion and *TPS1*, *TPS2* overexpression.
(DOC)

Table S2 Oligonucleotides used for quantitative RT-PCR.
(DOC)

## References

[1] Mussatto SI, Dragone G, Guimãr es PMR, Silva JPA, Carneiro LM (2010). Technological trends, global market, and challenges of bio-ethanol production.. Biotechnol Adv.

[2] Ingledew W (1999). Alcohol production by *Saccharomyces cerevisiae*: a yeast primer..

[3] Bai F, Anderson W, Moo-Young M (2008). Ethanol fermentation technologies from sugar and starch feedstocks.. Biotechnol Adv.

[4] Wang FQ, Gao CJ, Yang CY, Xu P (2007). Optimization of an ethanol production medium in very high gravity fermentation.. Biotechnol Lett.

[5] Zhao X, Bai F (2009). Mechanisms of yeast stress tolerance and its manipulation for efficient fuel ethanol production.. J Biotechnol.

[6] Dinh TN, Nagahisa K, Hirasawa T, Furusawa C, Shimizu H (2008). Adaptation of *Saccharomyces cerevis*iae cells to high ethanol concentration and changes in fatty acid composition of membrane and cell size.. PLoS One.

[7] Vianna CR, Silva CLC, Neves MJ, Rosa CA (2008). *Saccharomyces cerevisiae* strains from traditional fermentations of Brazilian cacha a: trehalose metabolism, heat and ethanol resistance.. Antonie Van Leeuwenhoek.

[8] Fiedurek J, Skowronek M, Gromada A (2011). Selection and adaptation of *Saccharomyces cerevisae* to increased ethanol tolerance and production.. Pol J Microbiol.

[9] Nissen TL, Kielland-Brandt MC, Nielsen J, Villadsen J (2000). Optimization of ethanol production in *Saccharomyces cerevisiae* by metabolic engineering of the ammonium assimilation.. Metab Eng.

[10] Gibson BR, Lawrence SJ, Leclaire JPR, Powell CD, Smart KA (2007). Yeast responses to stresses associated with industrial brewery handling.. FEMS Microbiol Rev.

[11] Wu H, Zheng X, Araki Y, Sahara H, Takagi H (2006). Global gene expression analysis of yeast cells during sake brewing.. Appl Environ Microbiol.

[12] Gong J, Zheng H, Wu Z, Chen T, Zhao X (2009). Genome shuffling: Progress and applications for phenotype improvement.. Biotechnol Adv.

[13] Otte B, Grunwaldt E, Mahmoud O, Jennewein S (2009). Genome shuffling in *Clostridium diolis* DSM 15410 for improved 1, 3-propanediol production.. Appl Environ Microbiol.

[14] Shi D, Wang C, Wang K (2009). Genome shuffling to improve thermotolerance, ethanol tolerance and ethanol productivity of *Saccharomyces cerevisiae*.. J Ind Microbiol Biotechnol.

[15] Hou L (2010). Improved Production of Ethanol by Novel Genome Shuffling in *Saccharomyces cerevisiae*.. Appl Biochem Biotechnol.

[16] Valadi H, Larsson C, Gustafsson L (1998). Improved ethanol production by glycerol-3-phosphate dehydrogenase mutants of *Saccharomyces cerevisiae*.. Appl Microbiol Biotechnol.

[17] Graves T, Narendranath NV, Dawson K, Power R (2007). Interaction effects of lactic acid and acetic acid at different temperatures on ethanol production by Saccharomyces cerevisiae in corn mash.. Appl Microbiol Biotechnol.

[18] Gietz RD, Schiestl RH (2007). High-efficiency yeast transformation using the LiAc/SS carrier DNA/PEG method.. Nat Protoc.

[19] Tan H, Fu L, Seno M (2010). Optimization of Bacterial Plasmid Transformation Using Nanomaterials Based on the Yoshida Effect.. Int J Mol Sci.

[20] Gueldener U, Heinisch J, Koehler G, Voss D, Hegemann J (2002). A second set of loxP marker cassettes for Cre-mediated multiple gene knockouts in budding yeast.. Nucleic Acids Res.

[21] Hou L (2009). Novel methods of genome shuffling in *Saccharomyces cerevisiae*.. Biotechnol Lett.

[22] Zheng DQ, Wu XC, Tao XL, Wang PM, Li P (2010). Screening and construction of *Saccharomyces cerevisiae* strains with improved multi-tolerance and bioethanol fermentation performance.. Bioresour Technol.

[23] Mizoguchi H, Hara S (1996). Effect of fatty acid saturation in membrane lipid bilayers on simple diffusion in the presence of ethanol at high concentrations.. J Ferment Bioeng.

[24] Belviso S, Bardi L, Bartolini AB, Marzona M (2004). Lipid nutrition of *Saccharomyces cerevisiae* in winemaking.. Can J Microbiol.

[25] Dong Y, Yang Q, Jia S, Qiao C (2007). Effects of high pressure on the accumulation of trehalose and glutathione in the *Saccharomyces cerevisiae* cells.. Biochem Eng J.

[26] Hottiger T, Schmutz P, Wiemken A (1987). Heat-induced accumulation and futile cycling of trehalose in *Saccharomyces cerevisiae*.. J Bacteriol.

[27] Jules M, Beltran G, François J, Parrou JL (2008). New insights into trehalose metabolism by *Saccharomyces cerevisiae*: *NTH2* encodes a functional cytosolic trehalase, and deletion of *TPS1* reveals Ath1p-dependent trehalose mobilization.. Appl Environ Microbiol.

[28] Jules M, Guillou V, François J, Parrou JL (2004). Two distinct pathways for trehalose assimilation in the yeast *Saccharomyces cerevisiae*.. Appl Environ Microbiol.

[29] Liang L, Wang X, Zhu K, Chi Z (2007). Trehalose synthesis in *Saccharomycopsis fibuligera* does not respond to stress treatments.. Appl Microbiol Biotechnol.

[30] Bradford MM (1976). A rapid and sensitive method for the quantitation of microgram quantities of protein utilizing the principle of protein-dye binding.. Anal Biochem.

[31] Teste MA, Duquenne M, François J, Parrou JL (2009). Validation of reference genes for quantitative expression analysis by real-time RT-PCR in *Saccharomyces cerevisiae*.. BMC Mol Biol.

[32] Argueso JL, Westmoreland J, Mieczkowski PA, Gawel M, Petes TD (2008). Double-strand breaks associated with repetitive DNA can reshape the genome.. Proc Natl Acad Sci U S A.

[33] Ding J, Huang X, Zhang L, Zhao N, Yang D (2009). Tolerance and stress response to ethanol in the yeast *Saccharomyces cerevisiae*.. Appl Microbiol Biotechnol.

[34] Lei J, Zhao X, Ge X, Bai F (2007). Ethanol tolerance and the variation of plasma membrane composition of yeast floc populations with different size distribution.. J Biotechnol.

[35] Mannazzu I, Angelozzi D, Belviso S, Budroni M, Farris GA (2008). Behaviour of *Saccharomyces cerevisiae* wine strains during adaptation to unfavourable conditions of fermentation on synthetic medium: cell lipid composition, membrane integrity, viability and fermentative activity.. Int J Food Microbiol.

[36] You KM, Rosenfield CL, Knipple DC (2003). Ethanol tolerance in the yeast *Saccharomyces cerevisiae* is dependent on cellular oleic acid content.. Appl Environ microbiol.

[37] Aguilera F, Peinado R, Millan C, Ortega J, Mauricio J (2006). Relationship between ethanol tolerance, H+-ATPase activity and the lipid composition of the plasma membrane in different wine yeast strains.. Int J Food Microbiol.

[38] Li LL, Ye YR, Pan L, Zhu Y, Zheng SP (2009). The induction of trehalose and glycerol in *Saccharomyces cerevisiae* in response to various stresses.. Biochem Biophys Res Commun.

[39] Chi Z, Arneborg N (1999). Relationship between lipid composition, frequency of ethanol-nduced respiratory deficient mutants, and ethanol tolerance in *Saccharomyces cerevisiae*.. J Appl Microbiol.

[40] Castillo Agudo L (1992). Lipid content of *Saccharomyces cerevisiae* strains with different degrees of ethanol tolerance.. Appl Microbiol Biotechnol.

[41] Kim HS, Kim NR, Choi W (2011). Total fatty acid content of the plasma membrane of *Saccharomyces cerevisiae* is more responsible for ethanol tolerance than the degree of unsaturation.. Biotechnol Lett.

[42] Shobayashi M, Mitsueda SI, Ago M, Fujii T, Iwashita K (2005). Effects of culture conditions on ergosterol biosynthesis by *Saccharomyces cerevisiae*.. Biosci Biotechnol Biochem.

[43] Kaino T, Takagi H (2008). Gene expression profiles and intracellular contents of stress protectants in *Saccharomyces cerevisiae* under ethanol and sorbitol stresses.. Appl Microbiol Biotechnol.

[44] Hallsworth JE (1998). Ethanol-induced water stress in yeast.. J Ferment Bioeng.

[45] Adams J, Puskas-Rozsa S, Simlar J, Wilke C (1992). Adaptation and major chromosomal changes in populations of *Saccharomyces cerevisiae*.. Curr Genet.

