# Supplementary material for: A Novel Strategy to Construct Yeast Saccharomyces cerevisiae Strains for Very High Gravity Fermentation
Source: PLoS One. 2012 Feb 17;7(2):e31235. doi: 10.1371/journal.pone.0031235 (PMC3281935; doi:10.1371/journal.pone.0031235)
Supplement: Figure S1 — Trehalose content and cell viability of strain Z5, Z5TPS1-2, SZ3-1 under 15% ethanol stress. (DOC) [file pone.0031235.s001.doc]

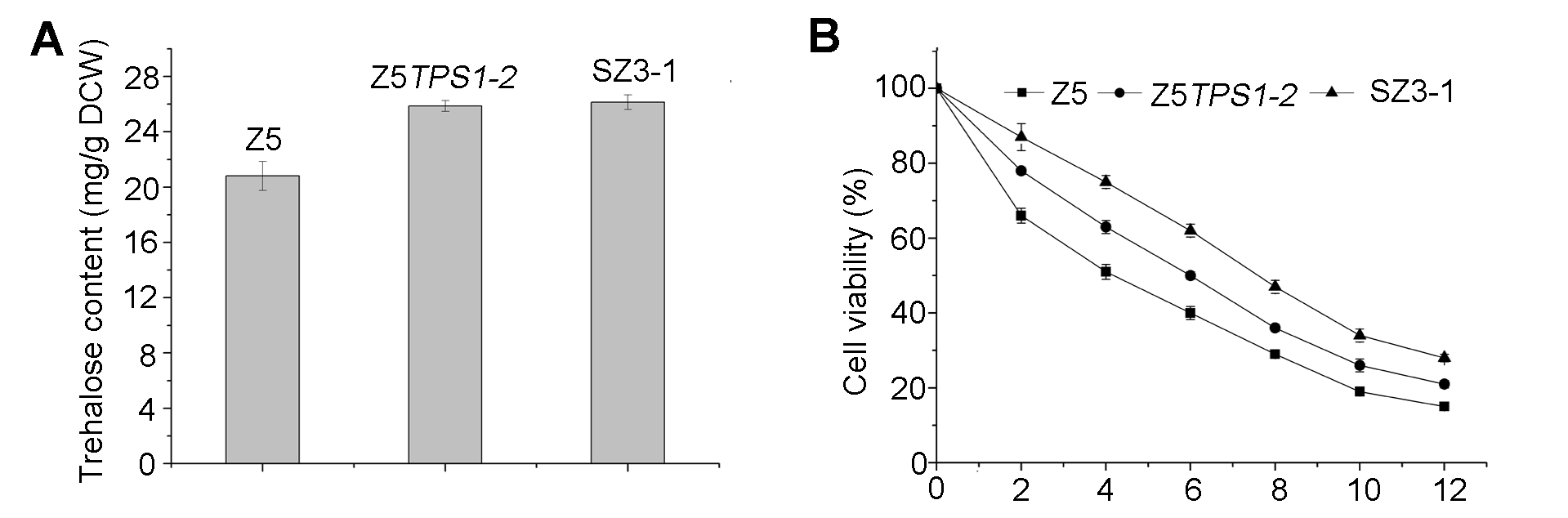


**Figure S1. Trehalose content and cell viability of strain Z5, Z5*TPS1-2*, SZ3-1 under 15% ethanol stress.** Yeast strains were harvested at the stationary phase and exposed to ethanol stress. Trehalose content (A) of each strain was measured after exposure to ethanol for two hours. Cell viability (B) for each strain was measured every two hours.
