# Supplementary material for: A Novel Strategy to Construct Yeast Saccharomyces cerevisiae Strains for Very High Gravity Fermentation
Source: PLoS One. 2012 Feb 17;7(2):e31235. doi: 10.1371/journal.pone.0031235 (PMC3281935; doi:10.1371/journal.pone.0031235)
Supplement: Figure S2 — Comparison of DNA content of strains Z5 (red) and SZ3-1 (black) using flow cytometry. (DOC) [file pone.0031235.s002.doc]

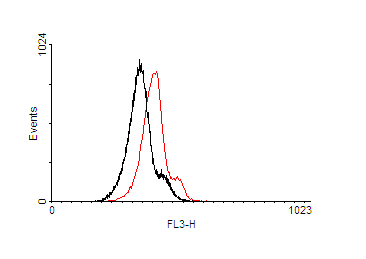


**Figure S2.** Comparison of DNA content of strains Z5 (red) and SZ3-1 (black) using flow cytometry. The stationary-phase cells of strain Z5 and SZ3-1 were fixed with 70% ethanol and stained with PI. DNA content was corresponded to the intensity of red fluorescenece cell emitted.
