# Supplementary material for: A Novel Strategy to Construct Yeast Saccharomyces cerevisiae Strains for Very High Gravity Fermentation
Source: PLoS One. 2012 Feb 17;7(2):e31235. doi: 10.1371/journal.pone.0031235 (PMC3281935; doi:10.1371/journal.pone.0031235)
Supplement: Table S1 — Primers used for GPD2 deletion and TPS1, TPS2 overexpression. (DOC) [file pone.0031235.s003.doc]

**Table S1.** Primers used for *GPD2* deletion and *TPS1*, *TPS2* overexpression

| Primer^a^ | Sequence (5’ to 3’) |
| --- | --- |
| GPD2US | GTTACCAGCTGCGGTTATTTTATCGGAACAT |
| GPD2UA | TTTAGTCGACTTTATTAAGGATCCTATAAGGAAGGGGAGCGAAGG |
| GPD2DS | ATTACCTGCAGGTCTGATCTTTCCTGTT |
| GPD2DA | CTTATCGGAATTCAATGGGGAGACAAGAT |
| JKS | ATCTGCCGGTCTCCCTATA |
| JKA | GCCAAACAAACTTTTCCC |
| GPD2S | TGCTATCCTCCTATGTTACTGA |
| GPD2A | TCCAAGGCTGACTTACCG |
| TPS1S | TATAAGGATCCATGACTACGGATAACGCT |
| TPS1A | CCTTCTCGAGACTTTCTAAAATGGCTAT |
| TPS2S | CTATAGATCTATGACCACCACTGCCCAA |
| TPS2A | ACGTCTCGAGTGCTGTTTTACTTTGCTA |

^a^Primer pairs GPD2US and GPD2UA, GPD2DS and GPD2DA were used to amplify fragment GPD2U and GPD2D respectively. Primer pairs JKS and JKA were confirmed primers for validation of correct insertion of *GPD2*-*kanMX*. Primer pairs GPD2S and GPD2A were used to determine whether *GPD2* gene was completely deleted. Primer TPS1S, TPS1A, TPS2S and TPS2A was used for *TPS1*, *TPS2* overexpression.
