# Supplementary material for: A Novel Strategy to Construct Yeast Saccharomyces cerevisiae Strains for Very High Gravity Fermentation
Source: PLoS One. 2012 Feb 17;7(2):e31235. doi: 10.1371/journal.pone.0031235 (PMC3281935; doi:10.1371/journal.pone.0031235)
Supplement: Table S2 — Oligonucleotides used for quantitative RT-PCR. (DOC) [file pone.0031235.s004.doc]

**Table S2.** Oligonucleotides used for quantitative RT-PCR

| Primer | Sequence(5’ to 3’) | Amplicon size (bp) | Efficiency^a^ |
| --- | --- | --- | --- |
| TPS1-F | AGGCTGGATTACATCAAAGG | 293 | 0.93 |
| TPS1-R | GTGGACGAGACCAAACAAAC |
| TPS2-F | ACGCCAAAGAACTGAAAGAA | 228 | 0.98 |
| TPS2-R | CATCACCCAAACATAATACA |
| TPS3-F | TAAACATCGGATTGATAGCG | 237 | 0.99 |
| TPS3-R | TTATGTGCGTAATGGCTTCC |
| TSL1-F | GTTAGTCTGAACGGTGTATGG | 293 | 1.04 |
| TSL1-R | TGAGCTGCCGATAAGGAAAG |
| ATH1-F | GAAGCAAGTCGCAACCAGTC | 137 | 1.00 |
| ATH1-R | ACTGCCACAACTATGTATCTT |
| NTH1-F | GGCATTATGGGCTGGACTTG | 214 | 0.97 |
| NTH1-R | ATAACCATAAGAACGGAGGC |
| ACT1-F | GGCTTCTTTGACTACCTTCCA | 89 | 1.09 |
| ACT1-R | AGAAACACTTGTGGTGAACGA |

^a^Efficiency(*E*) was determined using the formula *E*=10^(-1/slope)^-1,with the “slope” being the slope of the standard curve that was obtained from 10 fold serial dilution of sample cDNA.
